# Determination of speed and assessment of conditioning in horses submitted to a lactate minimum test—alternative approaches

**DOI:** 10.3389/fphys.2024.1324038

**Published:** 2024-04-25

**Authors:** Gabriel Vieira Ramos, Angélica Cristina Titotto, Guilherme Barbosa da Costa, Guilherme de Camargo Ferraz, José Corrêa de Lacerda-Neto

**Affiliations:** ^1^ Equine Sports Medicine Laboratory, Department of Veterinary Clinics and Surgery, School of Agrarian and Veterinary Sciences, Jaboticabal, Brazil; ^2^ Equine Exercise Physiology and Pharmacology Laboratory (LAFEQ), Department of Animal Morphology and Physiology, School of Agrarian and Veterinary Sciences, Jaboticabal, Brazil

**Keywords:** training, aerobic capacity, athletic horse, exercise test, AUC, angular method, LMS

## Abstract

The maximal lactate steady state (MLSS) is a well-known gold standard method for determining the aerobic capacity of athletic horses. Owing to its high cost and complex execution, there is a search for standardized exercise tests that can predict this value in a single session. One of the methods described for this purpose is the lactate minimum test (LMT), which could be more accurate despite being adequate to predict MLSS. This study aimed to examine the impact of training on the speed corresponding to lactate minimum speed (LMS) and to apply new mathematical methods to evaluate the fitness level of horses based on the curve obtained by the LMT. Ten Arabian horses underwent a 6-week training program based on LMS calculated by second-degree polynomial regression (LMS_P_). In addition, the LMS was also determined by visual inspection (LMS_V_), bi-segmented linear regression (LMS_BI_) and spline regression (LMS_S_). From the curve obtained during the LMT, it was possible to calculate angles α, β and ω, as well as the total area under the curve (AUC_TOTAL_) before (AUC_PRELMS_) and after (AUC_POSLMS_) the LMS. The methods for determining the LMS were evaluated by ANOVA, intraclass correlation coefficient (ICC) and effect size (ES) by Cohen’s d test. The Pearson correlation coefficient (r) between the proposed LMS determination methods and other mathematical methods was also calculated. Despite showing a good correlation (ICC >0.7), the LMS determination methods differed from each other (*p* < 0.05), albeit without a significant difference resulting from conditioning. There were reductions in α:β ratio, angle α, and AUC_POSTLMS_, with the latter indicating lower lactate accumulation in the incremental phase of LMT after conditioning, in addition to an improvement in the animals’ aerobic capacity. Considering that the most common methods for determining the LMS are applicable yet with low sensitivity for conditioning assessment, the approaches proposed herein can aid in analyzing the aerobic capacity of horses subjected to LMT. The mathematical models presented in this paper have the potential to be applied in human lactate-guided training program trials with a comparable study basis.

## 1 Introduction

Objective external training load prescription and aerobic fitness evaluation are essential in equestrian sports. Endurance performance tests are essential to define personalized loads for each type of sport and athletic profile in human and veterinary sports medicine (horses and dogs). Despite advancements in technology and research, much animal training, such as horse training, is still conducted based on empirical methods. These methods are often based on widespread protocols that are not tailored to each animal’s capabilities (Cunha et al., 2009; Ferasin and Marcora, 2009; Manzo et al., 2009; Alves et al., 2012; Rodrigues et al., 2017; Impellizzeri et al., 2019; Restan et al., 2019; De Maré et al., 2022). Lactate threshold (LT) is a key marker for running performance and lactate-guided conditioning program has become a cornerstone of endurance training (Dyson, 2002; Poole and Erickson, 2008; Singer et al., 2008; Peyré-Tartaruga and Coertjens, 2018; Impellizzeri et al., 2019). Scientists use standardized incremental exercise tests (IETs) to elicit an exponential rise in [Lac] and estimate the maximal lactate steady state (MLSS).

MLSS refers to the highest external load that can maintain a constant level of lactate concentration, with a variation of less than 1 mmol/L, during 30 min of exercise (Jamnick et al., 2020). Although considered the most reliable method to evaluate oxidative phosphorylation capacity, the MLSS requires a minimum of three consecutive tests on alternate days, which limits its practical application. Alternative tests that estimate the MLSS in a single exercise bout have been used. A literature review identified roughly 25 concepts used to analyze the lactate-speed curve (LSC) and describe some form of LT to predict MLSS (Faude et al., 2009). It should be emphasized that many equine studies have applied the established threshold concept of “onset of blood lactate accumulation” (OBLA), so-called fixed LT “V_4_,” the speed at which a plasma lactate concentration of 4 mmol/L. However, there has been ongoing debate surrounding this rigid cut-off value as it fails to consider individual differences in lactate kinetics. Besides, relying solely on the 4 mmol/L threshold could overestimate the MLSS (Lindner, 2010; Miranda et al., 2014; Soares et al., 2014; De Maré et al., 2022).

Alternatively, the lactate minimum test (LMT), which challenges an athlete’s lactate appearance-disappearance steady state like the MLSS test, could be performed. (Wahl et al., 2018). Such a test was first introduced into sports medicine in mid-1993 by (Tegtbur et al., 1993) and consists of three phases: (1) hyperlactatemia induction through a short-term high-intensity exercise; (2) dynamic recovery period to allow the diffusion of lactate produced by the muscles into the bloodstream; and (3) a typical incremental exercise bout. The U-shaped lactate-speed curve generated by the samples provides the speed that corresponds to the lowest [Lac] observed on the curve is called lactate minimum speed (LMS). It represents the equivalence between lactate production, redistribution, and wash-out (Messias et al., 2017b; De Maré et al., 2022). The speed that corresponds to the lowest [Lac] observed on the curve is called lactate minimum speed (LMS) and represents the equivalence between lactate production, redistribution and wash-out (Tegtbur et al., 1993). Since its introduction, the LMT has been used in several sports, including running, rowing, cycling, and swimming, to establish individualized training programs for elite athletes (Tegtbur et al., 1993; Bacon and Kern, 1999; Macintosh et al., 2002; Ribeiro et al., 2003; Sotero et al., 2009; Knoepfli-Lenzin and Boutellier, 2011; Campos et al., 2014).

Few trials have applied LMT to assess horse conditioning (Gondim et al., 2007; Miranda et al., 2014; Soares et al., 2014; De Maré et al., 2022). A study proposed profiling horses’ aerobic window (AW) in response to training using a modified LMT. This alternative mathematical method was performed for individually tailored AW assessment in standardbred mares (De Maré et al., 2022). Therefore, this study aims (1) to evaluate the effect of an LMS-based training program performed on a treadmill according to previously described methodologies and (2) to propose a new mathematical approaches (i.e., angular method (AM) and area under the curve (AUC)) as complementary methods for prescribing and assessing the horses conditioning based on the curve obtained by LMT.

## 2 Material and methods

### 2.1 Animals

Ten Arabian purebred (AP) horses (six geldings and four females) aged 9 ± 3 years belonging to the experimental herd of the School of Agrarian and Veterinary Sciences (FCAV), Unesp, Jaboticabal, Brazil, were used. The present study was approved by the Ethics Committee on the Use of Animals of the same institution (CEUA - FCAV) under protocol no. 005128/18. The inclusion criteria were: healthy AP horses, had not participated in any conditioning program for at least 12 months and were not undergoing drug treatment. Three weeks before the beginning of the experiment and during its course, the animals were housed in the Equine Farming Sector of the same institution on Tifton pastures measuring 100 × 70 m. They received 40% of the energy demand for moderate work (NRC, 2007) in commercial concentrate. Before the experimental study, the horses had their health assessed through complete physical examinations, blood count and cardiac evaluation by electrocardiogram and echocardiogram, all carried out at rest. The selected animals were included in the regular annual endo- and ectoparasite control program (Dorax Plus, Grupo União Química Farmacêutica S. A., Brazil) and vaccinated against respiratory viruses and tetanus (Lexington Gold, Dechra Brazil, Brazil).

### 2.2 Acclimatization to treadmill exercise

Before to the experimental period, the horses were acclimatized to the treadmill for 5 days. They were taken from the Equine Farming Sector to the premises of the Equine Sports Medicine Laboratory (LMEE), FCAV/UNESP—Jaboticabal campus. On the first day, the animals were led individually, with the halter on, in order to walk at their own pace on the treadmill belt (Galloper, Sahinco LTDA., Brazil), whose length is 5.5 m. At the end of the activity, the group of 10 horses walked on the treadmill in line, going around the building three times. On the second day, they were adapted to the girth and required to walk at their own pace again so that all of them passed over the belt with the treadmill turned off, also going around the building three times. On the third day, with the girth attached and secured to the safety belt, the horses were placed on the treadmill, which was then turned on at a speed that allowed them to walk at their own pace for 15 min. On the fourth day, the previous procedure was repeated, but at a higher speed in order to encourage trotting. On the fifth and last day of adaptation, the horses were induced to perform a collected gallop, both in the horizontal plane and at a 2% incline.

### 2.3 Lactate minimum test (LMT)

To determine the LMS, two LMTs were applied with a 6-week conditioning period in between. During each LMT, the ambient temperature and humidity were constantly monitored by a digital thermohygrometer (MTH-1360, Minipa do Brasil, Brazil) and controlled using air conditioning and a spray cooler, respectively. The applied LMT protocol was adapted from Soares et al. (2014) and consisted of three steps, namely, (I) hyperlactatemia induction, (II) active recovery, and (III) incremental exercise test (IET). Before the first step, the horses performed 10 min of warm-up divided into two steps of 5 min at a speed of 1.4 m/s and 3.5 m/s, respectively, with the treadmill in a horizontal position (0% incline). Hyperlactatemia began immediately after the warm-up period at a 6% incline and a speed of 10 m/s for 120 s. At the end of this phase, the animals performed an active recovery with the treadmill in a horizontal position and at a speed of 1.7 m/s for 120 s. Next, the IET started at a 6% incline and an initial speed of 3.0 m/s for 4 min and 27 s. Each subsequent increase was 0.5 m/s, that is, 3.5 m/s for 3 min and 49 s; 4.0 m/s for 3 min and 20 s; 4.5 m/s for 2 min and 58 s; 5.0 m/s for 2 min and 40 s; 5.5 m/s for 2 min and 26 s; 6.0 m/s for 2 min and 13 s; 6.5 m/s for 2 min and 3 s; 7.0 m/s for 1 min and 54 s; and 7.5 m/s for 1 min and 47 s. The relative times for each increase in speed were established in such a way that the horses covered approximately 800 m in each. Between each IET phase, the treadmill was turned off for 60 s for blood sample collection. The 1-min rest period between incremental exercise phases may have minimized the delay in lactate transport from muscles to the bloodstream. Immediately after the IET, the horses performed 10 min of cool-down divided into two steps of 5 min at 3.5 m/s and 1.7 m/s, respectively. The same LMT protocol was applied before and after 6 weeks of conditioning.

### 2.4 Sample collection and processing

Before the LMT, each animal underwent right jugular venous catheterization, performed aseptically using a 14G catheter (Angiocatt, BD Ind. Cirúrgicas Ltda., Brazil) coupled to a 120 cm extender (Medsonda, Ind. e Com. de Prod. Hosp. Desc. Ltda., Brazil) to facilitate blood collection, even if the horses were moving. The procedure was carried out in a room adjacent to the treadmill, which contains stocks with rubber floors. The region where the catheter was inserted was previously shaved, cleaned and desensitized by a local anaesthetic block with 3.0 mL of lidocaine 1% (Xylestesin, Cristália Prod. Químicos e Farmacêuticos Ltda., Brazil). Between the collections, the catheter and extender were filled with heparinized saline solution (1:50) in order to maintain the system patency. Before each collection, 20 mL of solution was aspirated and discarded. A total of 4 mL of blood samples was collected in two 3 mL blood gas syringes containing 80 U.I. of calcium-balanced lytic heparin (BD Vacutainer, BD Ind. Cirúrgicas Ltda., Brazil) at rest, after the hyperlactatemia period, at the end of active recovery, between each IET interval, immediately after the IET and 20 min after the cool-down period. After the last collection, the catheter was removed, and anti-inflammatory gel was applied to the site–a procedure that was repeated twice a day for 3 days. The blood samples were collected in syringes containing lytic heparin and immediately taken to the laboratory at the LMEE facilities, where they were promptly analyzed using a blood gas analysis device (Cobas b 123 Instrument with Auto QC, CO-Oximeter, Roche Diagnostic GmBH, Germany) to determine [Lac].

### 2.5 Training

**FIGURE 1:**
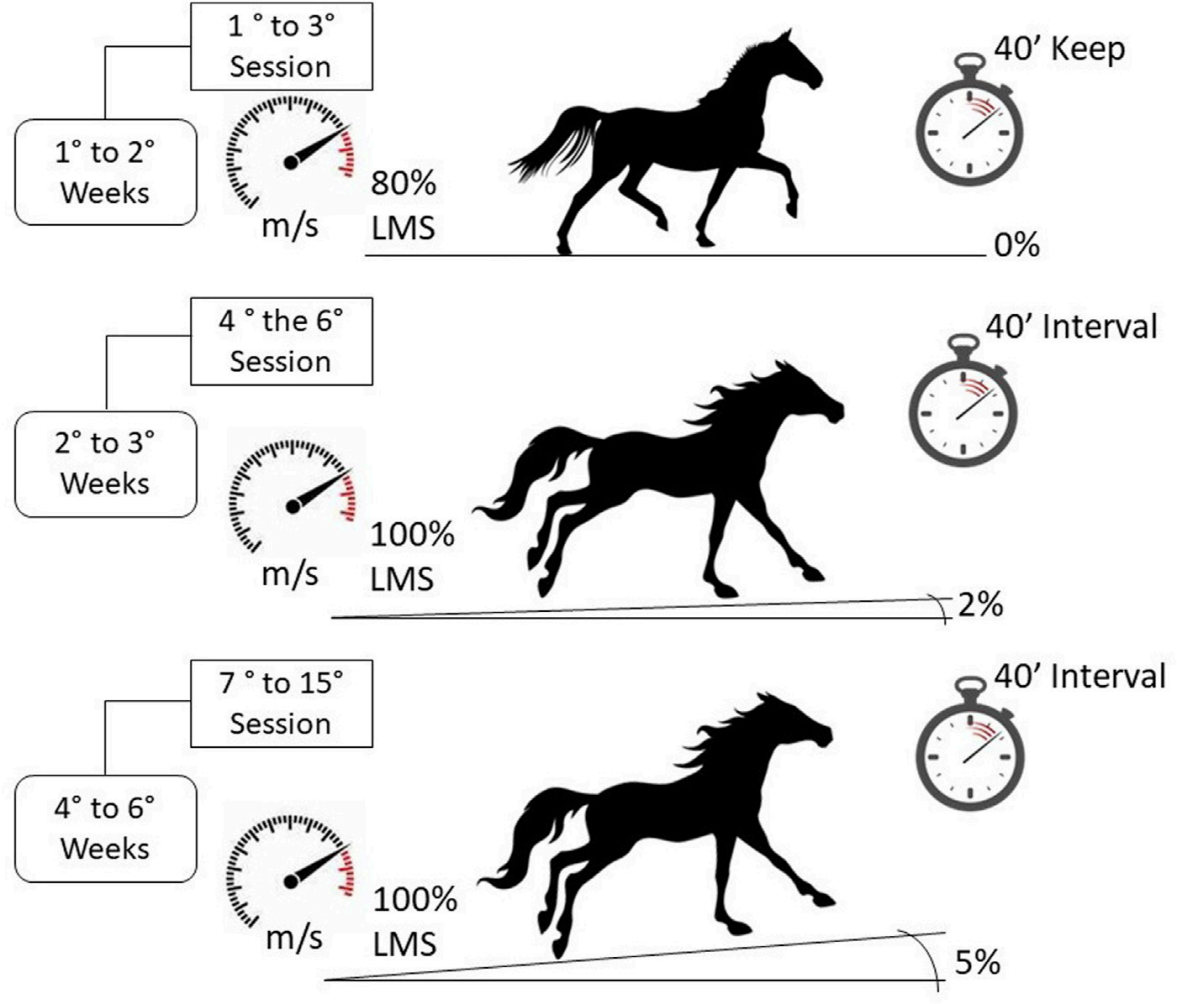
Schematic representation of the training protocol used according to the sessions carried out. LMS: lactate minimum speed.

The speed for conditioning was calculated individually based on the LMS determined by second-degree polynomial regression (LMS_P_) during the first LMT. The training lasted for 6 weeks, during which the animals were trained for five bi-weekly sessions of 40 min each, on alternate days, totaling 15 sessions. The training program was the same as that used by Santos et al. (2022), implemented progressively, as represented in Figure 1.

### 2.6 Determination of the speed ​​corresponding to the lactate minimum speed (LMS)

**FIGURE 2:**
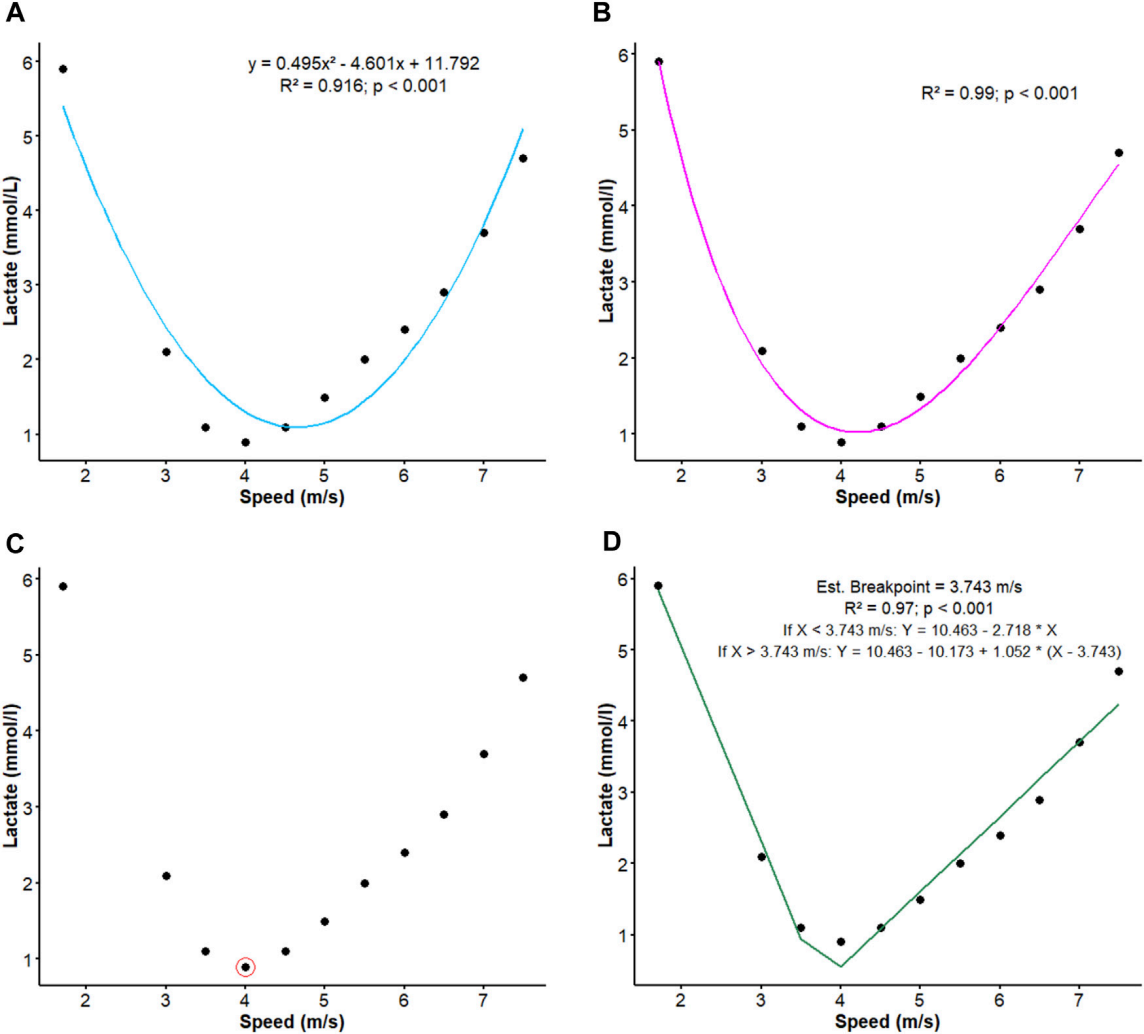
Different methods used to determine the speed corresponding to lactate minimum speed (LMS) obtained during the lactate minimum test (LMT). LMS calculated by **(A)** second-degree polynomial regression (LMS_P_), **(B)** spline regression (LMS_S_), **(C)** visual inspection (LMS_V_) and **(D)** bi-segmented regression (LMS_BI_).

The speed corresponding to lactate minimum speed was determined by four different methods: second-degree polynomial regression (LMS_P_), spline regression (LMS_P_), visual inspection (LMS_V_) and bi-segmented regression (LMS_BI_) (Figure 2).

#### 2.6.1 Second-degree polynomial regression (LMS_P_)

This method constitutes a type of linear regression adjusted to the dataset that presents a curvilinear relationship between the dependent (explanatory) and independent (predictor) variables. To determine lactate concentrations (dependent variable) as a function of speed (independent variable), we applied a quadratic equation (y = ax^2^ + bx + c), which defines the second-degree polynomial regression (Figure 2A). According to Restan et al. (2019) and Ferraz et al. (2022), the determination of LMS_P_ has superior reliability compared to other previously described methods and, therefore, was chosen to guide the training.

#### 2.6.2 Spline regression (LMS_S_)

In spline regression, curves are assumed to be in the form of f(X), with only one predictor variable for each explanatory variable. When a series of knots τ1 < τ2 < τ3 … < τK is defined in the interval of X, polynomials of degree d are arbitrarily established for each segment between the knots (Figure 2B). In this study, we utilized B-spline, a type of spline with a basis consisting of a cubic spline parameterization. The basis is based on a series of knots:

$$\xi_{1}\leq ...\;\xi_{d}\leq \xi_{d+1}\;<\;\xi_{d+2\;}<\;\ldots \;<{\;\xi }_{d+K+1\;}<\;\xi_{d+K+2}\leq \xi_{d+K+3\;}\leq \ldots \leq \xi_{2d+K+2}$$

where the inner knots $\tau_{1},\tau_{2},\tau_{3}\;\ldots \;\tau_{K}$ are defined by the expression $\xi_{d+2}∶=\tau_{1},\ldots ,\xi_{d+K+1}∶=\tau_{K}$ , while the so-called boundary knots are defined by $\xi_{d+1}∶=a,\xi_{d+K+2}∶=b$ . For the present study, the B-spline created was limited according to the data range, causing the additional knots to be equal to the boundary knots (Perperoglou et al., 2019).

#### 2.6.3 Visual inspection (LMS_V_)

Visual inspection was also carried out by three evaluators specialized in exercise physiology and with experience in this type of methodology in order to determine the speed corresponding to lactate minimum speed (Figure 2C). Since the determination of LMS_V_ was made after the second LMT, the evaluators were blind in relation to the animal evaluated and the moment at which the curve was determined (pre- or post-conditioning).

#### 2.6.4 Bi-segmented linear regression (LMS_BI_)

Considering the characteristic U-shaped curve observed during the active recovery and incremental phases, the bi-segmented regression model was applied to determine the breakpoint that coincides with the lowest [Lac] reached during the LMT and thus calculate the LMS (Figure 2D). In the present study, the breakpoint was estimated from a known initial value using the equation below:

$$\beta_{1}+\beta_{2}{\left(z_{1}-\tilde{\psi }\right)}_{+}+\gamma I{\left(z_{i}>\tilde{\psi }\right)}^{-}$$

where $\beta_{1}$ is the incline, $\beta_{2}$ is the difference between inclines and ψ is the breakpoint. As demonstrated by the equation, the parameter γ can be interpreted as a re-parameterization of ψ and contributes to the breakpoint estimation (Muggeo, 2008). Before the application of the bi-segmented model, the Davies test was used to evaluate differences between inclines and initially estimate the breakpoint. According to this test, the null hypothesis $H_{0}:\beta_{2}=0$ indicates the absence of a breakpoint since the differences between inclines are equal to zero (Muggeo, 2008).

### 2.7 Area under curve (AUC)

**FIGURE 3:**
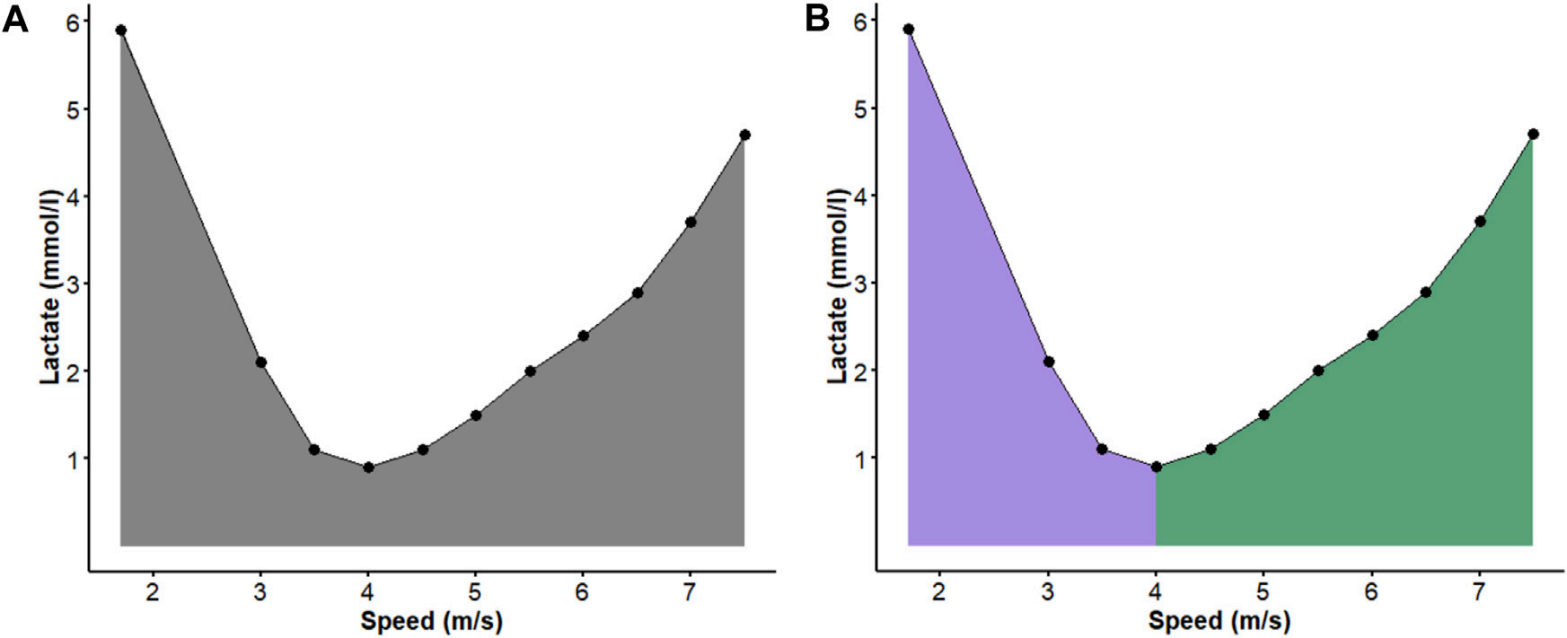
Representation of the area under curve (AUC) referring to the data obtained during the lactate minimum test (LMT) of an animal. For each animal, **(A)** the total AUC (AUC_TOTAL_) and **(B)** the AUC before (AUC_PRELMS_, lilac area) and after (AUC_POSTLMS_, green area) the speed corresponding to lactate minimum speed (LMS) obtained during the LMT were calculated.

Calculating the area under the curve (AUC) between two numerical variables can reduce a series of datasets to a single value representing the interaction between the two variables analyzed (Figure 3). In the present study, the determination of total AUC, i.e., the value calculated for all points of the lactate-speed curve obtained during the active recovery and IET phases (AUC_TOTAL_) (Figure 3A) represents, in general, the metabolic capacity of the animal in moments of predominance of aerobic metabolism (pre-LMS) and the association between aerobic and anaerobic metabolism (post-LMS). Therefore, in addition to AUC_TOTAL_, the areas before (AUC_PRELMS_) and after LMS (AUC_POSTLMS_) were calculated to evaluate the different metabolic profiles that make up the LMT (Figure 3B). Usually, AUC can be determined by the following expression:

$${AUC}_{0}^{t_{n}}=\int_{0}^{t_{n}}C\left(t\right)\;dt\approx \sum_{i=1}^{n-1}\int_{t_{i}}^{t_{i}+1}g\left(t,a_{i},b_{i}\ldots \right)dt$$

where $C_{i}$ is the concentration measured at certain times, $t_{i}\left(i=1,2\;\ldots \right)$ , and g is the interpolation function to be used. In the present study, natural cubic spline interpolation was used, resulting in:

$${AUC}_{0}^{t_{n}}\approx \sum_{i=1}^{n-1}\left\{\frac{h}{2}\left(C_{i}+C_{i+1}\right)-\frac{h^{3}}{24}(C_{i}^{n}+C_{i+1}^{n}\right\}$$

where $h=t_{i+1}-t_{i}$ (Purves, 1992).

### 2.8 Trigonometric method for angle determination

**FIGURE 4:**
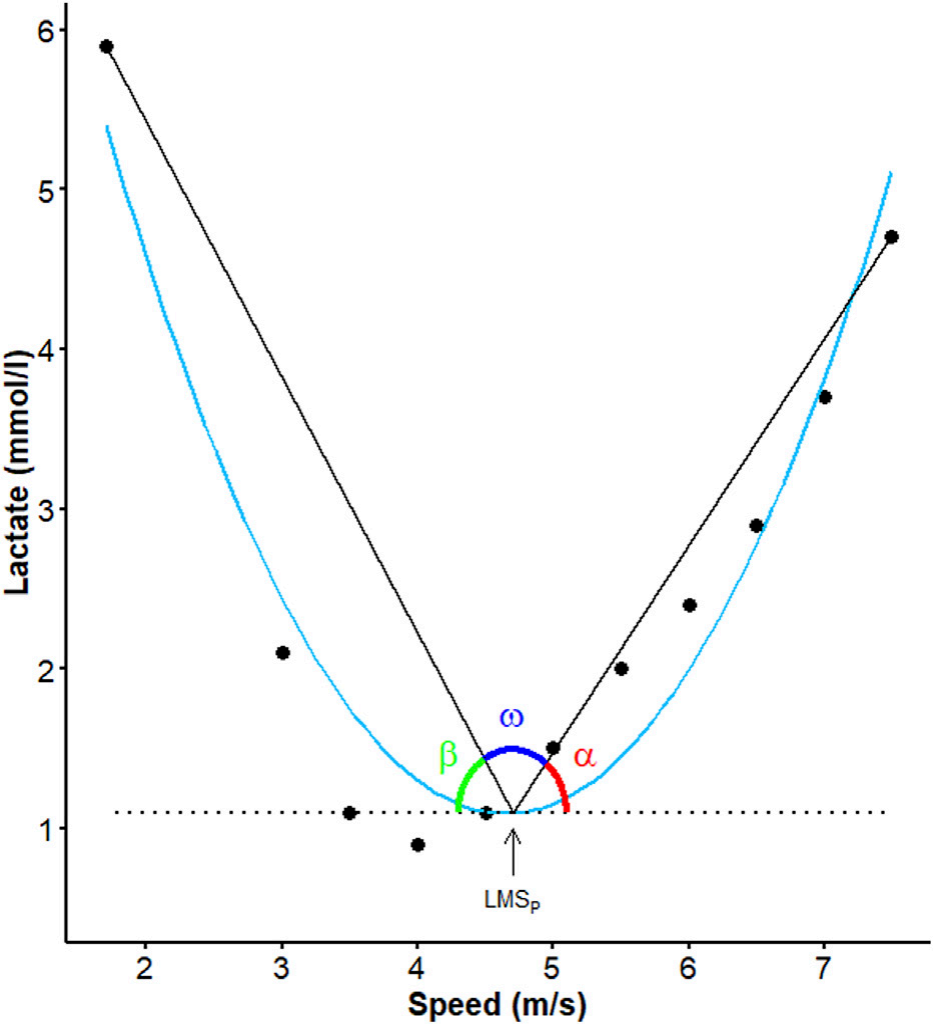
Curve obtained after the lactate minimum test of an animal representing the angles calculated from the speed corresponding to lactate minimum speed (arrow) individually determined by second degree polynomial regression (blue line).

The method used for calculating the aerobic window (AW) was derived from the angular approach described by De Maré et al. (2022), utilizing a trigonometric calculator in GeoGebra (version 6.0.826.0). First, a horizontal straight line was drawn from the LMS_P_ to the initial and final points of the curve, followed by the measurement of the angles β and α, respectively (as shown in Figure 4). Once these values were obtained, the angle ω was calculated by subtracting the sum of α and β from 180.

### 2.9 Statistical analysis

Regarding descriptive statistics, the parametric data were expressed as mean ± SD, while non-parametric data were presented as median, first and third quartiles. To compare the LMS_P_, LMS_S_, LMS_V_ and LMS_BI_ methods, analysis of variance for repeated measures (ANOVA) was applied with *post hoc* evaluation using the Tukey test. The interclass correlation coefficients (ICC) and Cohen’s d (effect size, ES) between the LMS_P_, LMS_S_, and other LMS determination methods were calculated. The correlation coefficients between the LMS determination methods and other proposed mathematical approaches and among them were also calculated using Pearson correlation. The interpretation of correlation coefficients (r) was carried out in accordance with Mukaka, (2012). Finally, the agreement among the LMS determination methods was evaluated using the graphical analysis proposed by Bland and Altman (1999) at a 95% confidence level. All analyses were performed using RStudio software (v. 2023.06.0 + 421″Mountain Hydrangea” for Windows).

## 3 Results

**FIGURE 5:**
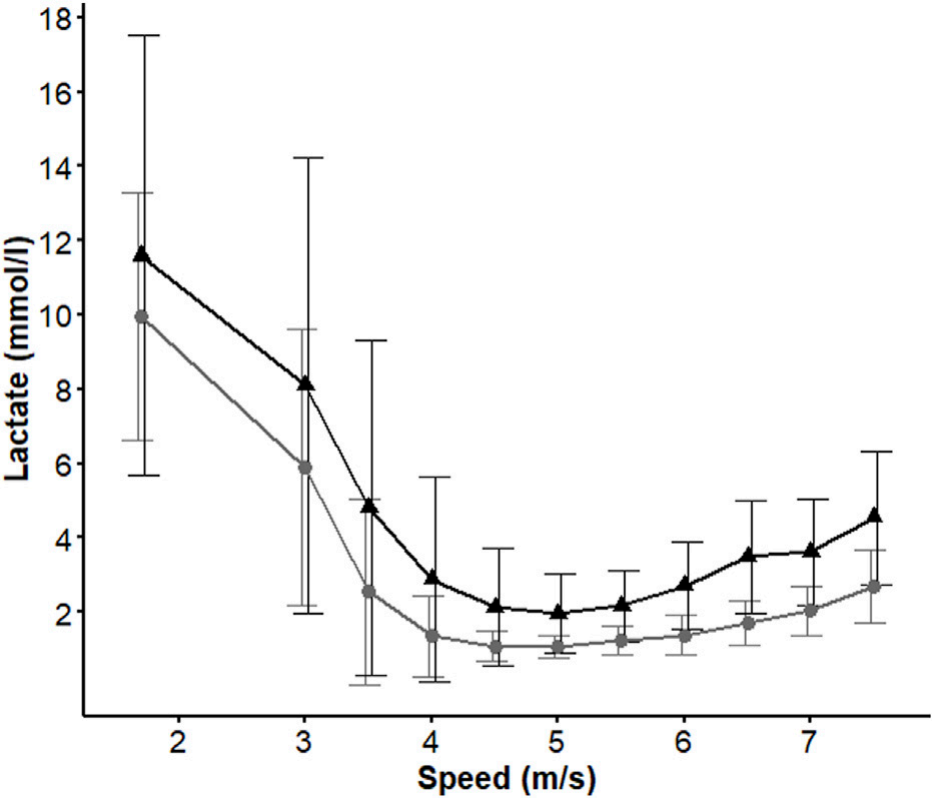
Mean lactate concentration values ​​during the lactate minimum test obtained before (▲) and after (●) conditioning guided by the speed corresponding to lactate minimum speed determined by second degree polynomial regression.

Table 1 displays the LMS values for each horse and Figure 5 shows the mean [Lac] values before and after the conditioning period. The differences found among the LMS determination methods were correlated with the highest speeds obtained by the polynomial method (LMS_P_: 5.456 m/s; *p* < 0.05) and the lowest speeds determined by the bi-segmented method (LMS_BI_: 4.175 m/s; *p* < 0.05). The LMS_S_ and LMS_V_ values, on the other hand, did not differ from each other (4.831 m/s and 4.625 m/s, respectively; *p* > 0.05).

**TABLE 1:** Individual values (C1 to C8) of the speed corresponding to lactate minimum speed (LMS) calculated by different methods before (pre-training) and after physical conditioning (post-training).

	Pre-training				Post-training			
	LMS_P_ (m/s)	LMS_S_ (m/s)	LMS_V_ (m/s)	LMS_BI_ (m/s)	LMS_P_ (m/s)	LMS_S_ (m/s)	LMS_V_ (m/s)	LMS_BI_ (m/s)
**C1**	4.7	4.2	4	3.743	5.5	4.8	4.5	4.141
**C2**	5.1	4.7	4	4.283	5.4	4.7	4.2	3.892
**C3**	6.1	5.4	5.5	5.136	5.4	4.8	4.9	3.64
**C4**	6.1	5.5	5	4.879	6	5.3	5.3	4.808
**C5**	5.5	4.8	4.5	4.879	5.5	4.8	4.7	3.566
**C6**	5.6	4.8	4.5	4.302	5.4	4.7	4.3	3.903
**C7**	4.7	4.3	4.3	4.13	5.4	4.9	4.8	4.121
**C8**	5.4	4.7	4.7	3.6	5.5	4.9	4.8	3.784

LMS_P_: LMS, obtained by second-degree polynomial regression; LMS_S_: LMS, obtained by spline regression; LMS_V_: LMS, determined by visual inspection; LMS_BI_: LMS, obtained by bi-segmented regression; m/s: meters per second.

All *p* values in bold indicate significant difference at 5%.

**FIGURE 6:**
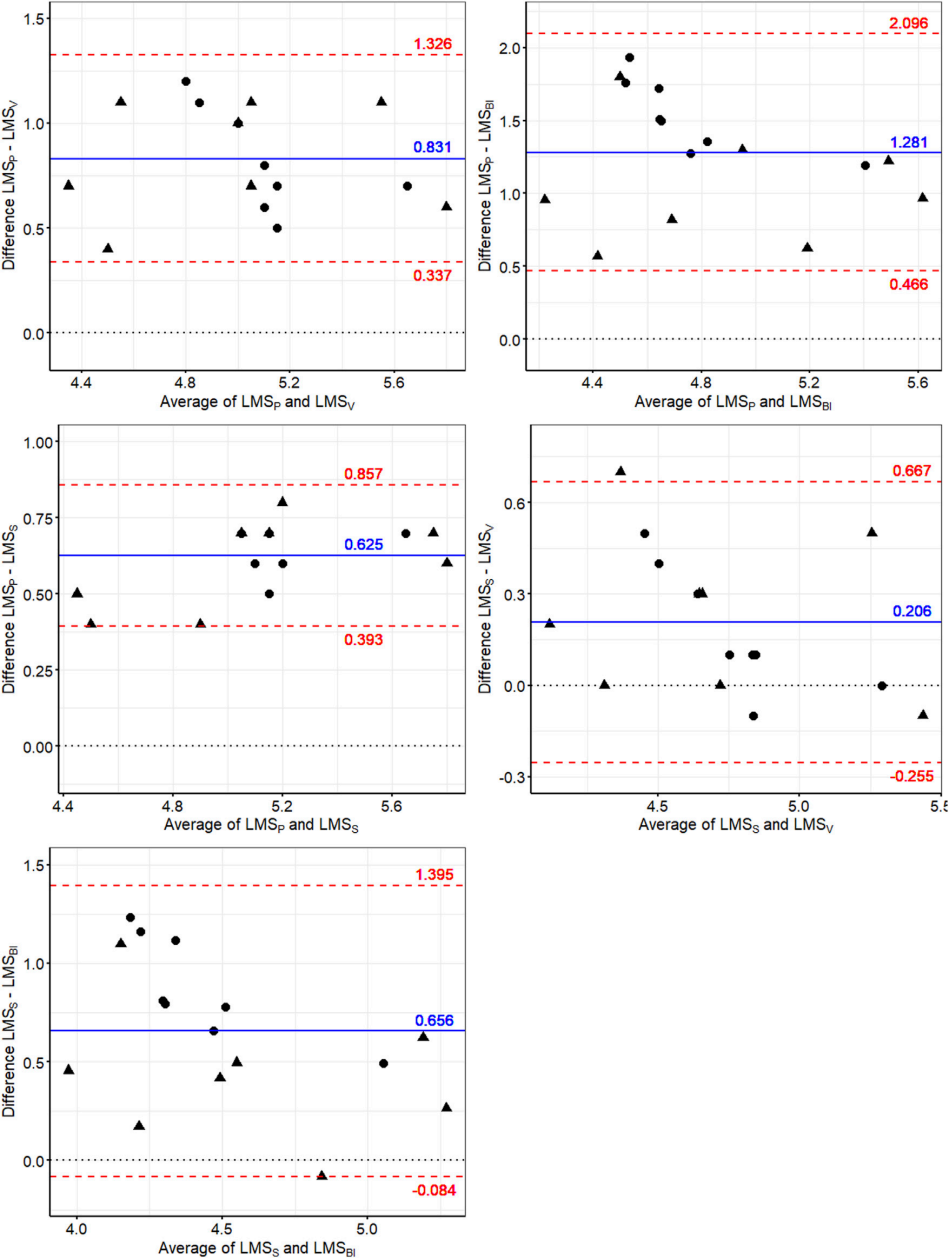
Bland-Altman plots to evaluate the agreement between the different methods used to determine the speed corresponding to lactate minimum speed (LMS) before (▲) and after (●) conditioning. The blue line corresponds to the mean bias between the respective methods, the upper and lower dashed red lines represent the upper (mean + 1.96*SD) and lower (mean—1.96*SD) limits of agreement, respectively, and the dotted line highlights the mean bias equal to 0. LMS_S_: LMS obtained by spline regression; LMS_V_: LMS determined by visual inspection; LMS_BI_: LMS obtained by bi-segmented regression.

Figure 6 shows the Bland-Altman plots used to evaluate the agreement among the LMS determination methods. As it can be observed, the smallest mean biases were between LMS_S_ and LMS_V_ (0.206 ± 0.235 m/s), LMS_P_ and LMS_S_ (0.625 ± 0.118 m/s), and LMS_S_ and LMS_BI_ (0.656 ± 0.377 m/s). Furthermore, the lowest limits of agreement were between LMS_P_ and LMS_S_. In contrast, the highest mean bias was between LMS_P_ and LMS_BI_ (1.281 ± 0.416 m/s). The ICC and EF values between LMS_P_ and LMS_S_ and among the other LMS determination methods can be found in Table 2. The high ICC value obtained between LMS_P_ and LMS_S_ indicates excellent agreement (ICC >0.9).

**TABLE 2:** Values of intraclass correlation coefficient (ICC) (lower and upper limits) and effect size (ES) (95% confidence interval) between the speed corresponding to lactate minimum speed (LMS) obtained by second-degree polynomial regression (LMS_P_) and spline regression (LMS_S_) and other LMS determination methods.

	LMS_P_	LMS_S_
**LMS**_**S**_	ICC: 0.97 (0.927–0.99)	-
	ES: 1.672 (0.833–2.51)	
**LMS**_**V**_	ICC: 0.9 (0.709–0.9)	ICC: 0.9 (0.71–0.96)
	ES: 2.002 (1.118–2.887)	ES: 0.533 (−0.201–1.268)
**LMS**_**BI**_	ICC: 0.74 (0.254–0.909)	ICC: 0.764 (0.32–0.92)
	ES: 2.801 (1.785–3.818)	ES: 1.52 (0.7–2.34)

LMS_V_: LMS, determined by visual inspection; LMS_BI_: LMS, obtained by bi-segmented regression.

All *p* values in bold indicate significant difference at 5%.

**FIGURE 7:**
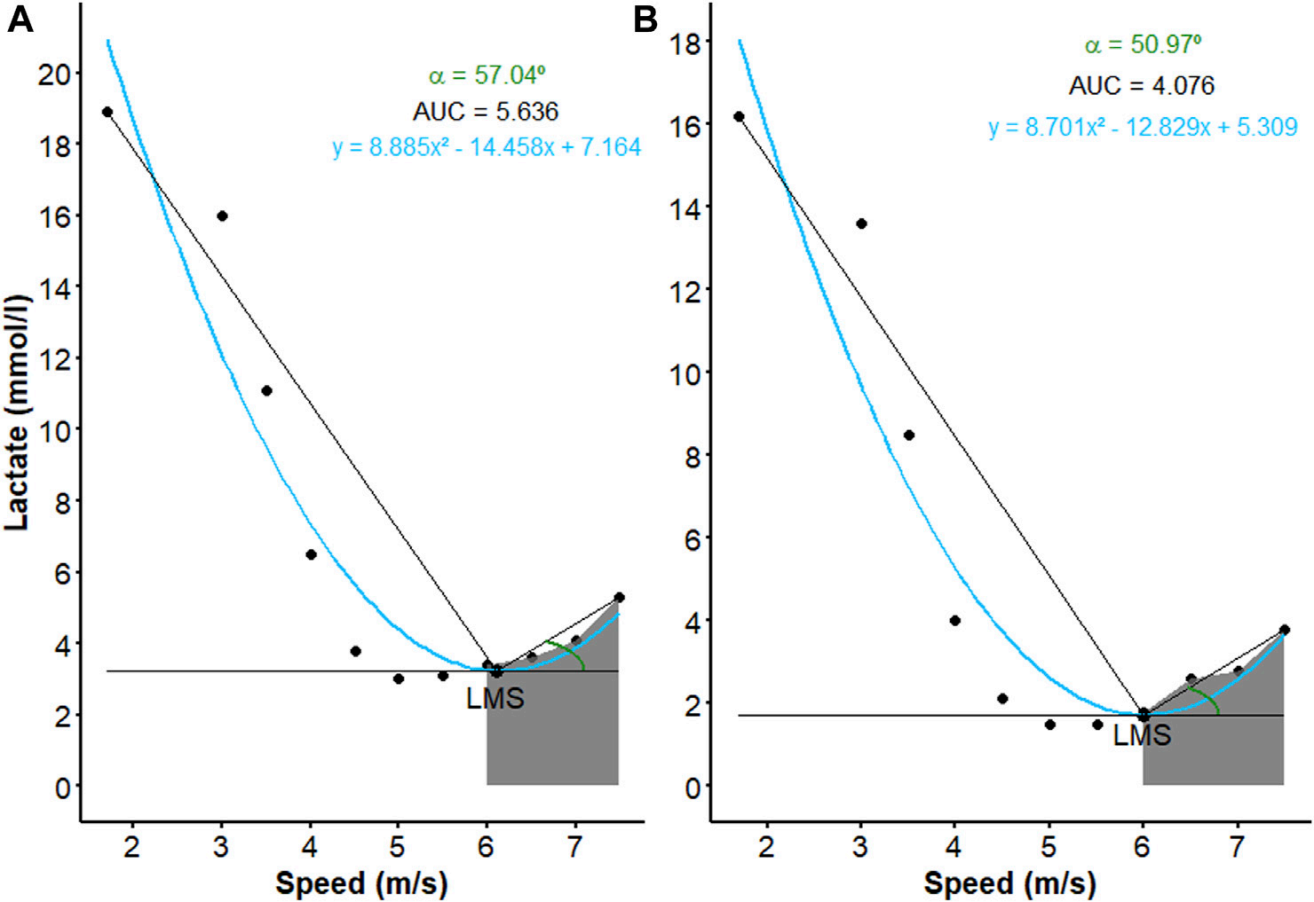
Curves obtained from the lactate minimum test (LMT) of an animal representing the reductions in angle α and area under curve (AUC, gray region) calculated after the speed corresponding to lactate minimum speed (LMS) determined by second polynomial regression degree (blue line and equation) during the lactate minimum test performed before **(A)** and after **(B)** 6 weeks of conditioning.

**FIGURE 8:**
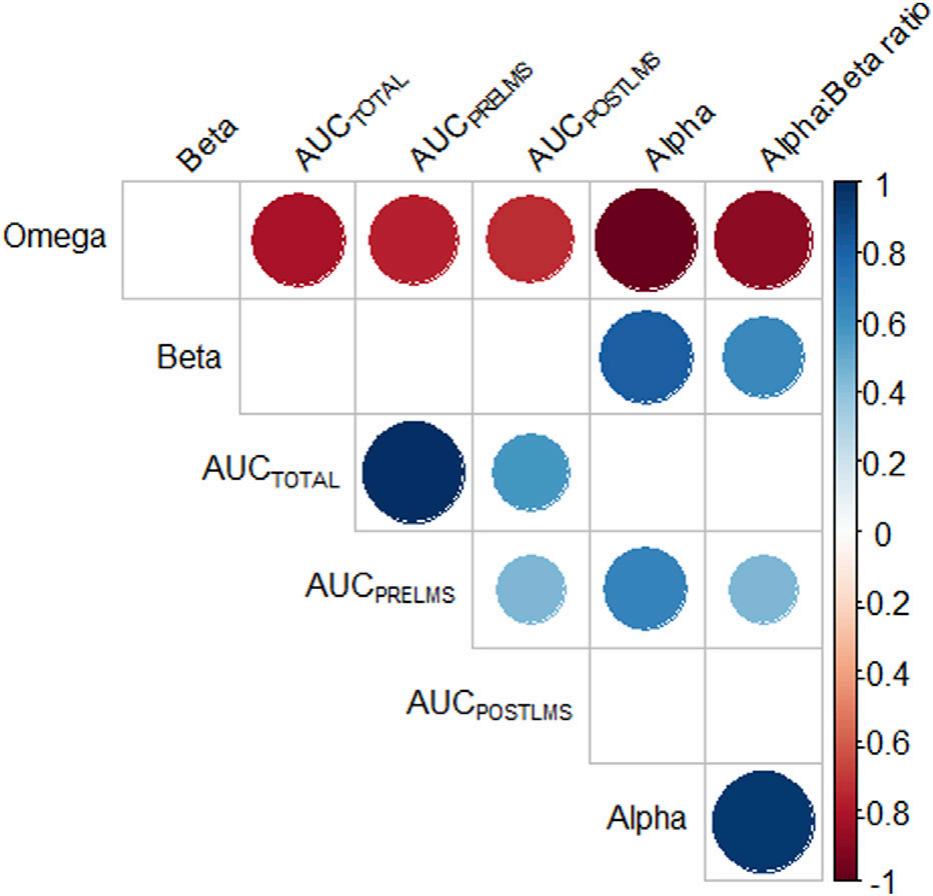
Correlogram between the variables proposed to evaluate the curve obtained from the lactate minimum test (LMT) performed on a treadmill. Regions with circles indicate correlations at a significance level of 5%, while blank regions indicate *p* values > 0.05. AUC_TOTAL_: total area under curve (AUC); AUC_PRELMS_: AUC that comprises active recovery and the beginning of incremental exercise test (IET) until LMS; AUC_POSTLMS_: AUC that comprises the end of IET, from LMS to the end of incremental phase.

Table 3 compares the analyzed variables before and after the conditioning period. As it can be seen, there were significant differences among the values of α, α:β ratio and AUC_POSTLMS_ after the conditioning period, as illustrated in Figure 7. The correlations between the LMS determination methods and the new approaches used herein are listed in Table 4. Among all the significant correlations, the r values were higher for the LMS_S_ method, with the exception of AUC_POSTLMS_:AUC_PRELMS_, whose value was higher than LMS_P_. Figure 8 illustrates the correlations among the new approaches proposed. According to this figure, there were high positive correlations between angles α and β (r = 0.818, *p* < 0.001), α and α:β ratio (r = 0.96; *p* < 0.001), α and β in relation to AUC_TOTAL_ (r = 0.73; *p* < 0.001 and r = 0.87; *p* < 0.001, respectively), β and AUC_PRELMS_ (r = 0.862; r < 0.001), α and AUC_POSLMS_ (r = 0.79; *p* < 0.001), and α:β ratio and AUC_POSLMS_ (r = 0.79; *p* < 0.001), while moderate correlations were found between α:β ratio and AUC_TOTAL_ (r = 0.538; *p* < 0.001), α and AUC_PRELMS_ (r = 0.66; *p* < 0.001), and β and AUC_POSLMS_ (r = 0.536; *p* < 0.001). Angle ω, on the other hand, presented very high negative correlations with α (r = −0.98; *p* < 0.001) and β (r = 0.92; *p* < 0.001) and high correlations with α:β ratio (r = −0.88; *p* < 0.001), AUC_TOTAL_ (r = −0.81; *p* < 0.001), AUC_PRELMS_ (r = −0.76; *p* < 0.001), and A AUC_POSTLMS_ (r = −0.73; *p* < 0.001). Lastly, AUC_TOTAL_ showed a high positive correlation with AUC_PRELMS_ (r = 0.987; *p* < 0.001) and a moderate correlation with AUC_POSLMS_ (r = 0.577; *p* = 0.02). The correlations between AUC_PRELMS_ and AUC_POSLMS_, and AUC_PRELMS_ and α:β ratio were not significant (*p* > 0.05). Two animals presented lameness during the IET and were withdrawn from the experiment without completing the experimental protocol.

**TABLE 3:** Means ± standard deviation of the speed corresponding to lactate minimum speed (LMS) obtained by different methods and other variables used in the analysis of the curve obtained during the lactate minimum test (LMT) carried out on a treadmill.

Variable	Pre-training	Post-training	p
**LMS**_**P**_ **(m/s)**	5.400 ± 0.548	5.513 ± 0.203	*0.4982*
**LMS**_**S**_ **(m/s)**	4.800 ± 0.460	4.862 ± 0.192	0.6744
**LMS**_**V**_ **(m/s)**	4.562 ± 0.507	4.688 ± 0.352	0.3703
**LMS**_**BI**_ **(m/s)**	4.369 ± 0.556	3.982 ± 0.391	0.1533
**α (°)**	47.481 ± 16.331	35.919 ± 12.669	**0.04536**
**β (°)**	65.951 ± 10.064	64.010 ± 6.392	0.6422
**ω (°)**	63.225 ± 25.442	80.075 ± 18.366	0.1434
**α:β ratio**	0.708 ± 0.208	0.551 ± 0.162	**0.03113**
**AUC**_**TOTAL**_	28.387 ± 16.912	19.228 ± 9.011	*0.25*
**AUC**_**PRELMS**_	21.651 ± 15.417	15.754 ± 8.221	*0.3828*
**AUC**_**POSTLMS**_	6.360 ± 2.303	3.477 ± 1.106	***0.01563***
**AUC**_**POSTLMS**_ **:AUC**_**PRELMS**_ **ratio**	0.445 ± 0.348	0.239 ± 0.05	*0.1484*

LMS_P_: LMS, obtained by second-degree polynomial regression; LMS_S_: LMS, obtained by spline regression; LMS_V_: LMS, determined by visual inspection; LMS_BI_: LMS, obtained by bi-segmented regression; α: alpha angle; β: beta angle; ω: omega angle; AUC_TOTAL_: total area under curve (AUC); AUC_PRELMS_: AUC, that comprises active recovery and the beginning of incremental exercise test (IET) until LMS; AUC_POSTLMS_: AUC, that comprises the end of IET, from LMS, to the end of incremental phase; AUC_PRELMS_: AUC_POSTLMS_, ratio: ratio of AUC_POSTLMS_, to AUC_PRELMS_. *P* and *p* values in italics were obtained by Student’s T and Wilcoxon tests, respectively. All *p* values in bold (*p* or *P* in italics) indicate a significant difference at 5%.

**TABLE 4:** Pearson correlation coefficients (r) and *p* values calculated between the speed corresponding to lactate minimum speed (LMS) obtained by second-degree polynomial regression (LMS_P_) and spline regression (LMS_S_) and other variables used to analyze the curve obtained during the lactate minimum test (LMT).

Variable	LMS_P_		LMS_S_		LMS_V_		LMS_BI_	
	r	p	r	p	r	p	r	p
**α (°)**	0.133	0.622	0.251	0.349	0.02	0.94	**0.719**	**0.002**
**β (°)**	**0.551**	**0.027**	**0.589**	**0.016**	0.249	0.352	**0.788**	**<0.001**
**ω (°)**	−0.292	0.273	−0.385	0.14	−0.104	0.7	**−0.776**	**< 0.001**
**α:β ratio**	−0.105	0.698	0.028	0.919	−0.121	0.656	**0.574**	**0.02**
**AUC**_**TOTAL**_	**0.685**	**0.003**	**0.737**	**0.001**	**0.514**	**0.042**	**0.836**	**< 0.001**
**AUC**_**PRELMS**_	**0.783**	**< 0.001**	**0.824**	**< 0.001**	**0.592**	**0.016**	**0.834**	**< 0.001**
**AUC**_**POSTLMS**_	−0.187	0.487	−0.099	0.714	0.24	0.371	0.428	0.098
**AUC**_**POSTLMS**_ **:AUC**_**PRELMS**_	**−0.866**	**< 0.001**	**−0.805**	**< 0.001**	**−0.615**	**0.011**	−0.333	0.206

LMS_V_: LMS, determined by visual inspection; LMS_BI_: LMS, obtained by bi-segmented regression; α: alpha angle; β: beta angle; ω: omega angle; AUC_TOTAL_: total area under curve (AUC); AUC_PRELMS_: AUC, that comprises active recovery and the beginning of incremental exercise test (IET) until LMS; AUC_POSTLMS_: AUC, that comprises the end of IET, from LMS, to the end of incremental phase; AUC_PRELMS_: AUC_POSTLMS_, ratio: ratio of AUC_POSTLMS_, to AUC_PRELMS_. *p* values ​​in bold indicate a significance level of 5%.

## 4 Discussion

We hypothesized that diagnosing endurance performance is possible using unconventional methods based on the LMT procedure. The main contribuition of the current research was that the alpha angle, alpha:beta ratio, and AUC_POSTLMS_, variables obtained from trigonometric and area under curve (AUC) methods, were able to detect an increase in aerobic fitness in Arabian horses. This study utilized Arabian horses owing to their suitable morphological, energetic metabolism, muscular features for endurance races (Cottin et al., 2010). Before and during the trial, the horses were given the freedom to exercise naturally on pasture. This management approach provides the horses with a voluntary exercise that is inherent to their physiology. The LMT protocol has proven to be both effective and safe in determining the LMS. This protocol allows for obtaining a “U" curve, which is necessary for determining the LMS. This traditional protocol has been studied as an option for predicting MLSS in humans (Macintosh et al., 2002; Dotan et al., 2011; Knoepfli-Lenzin and Boutellier, 2011; Wahl et al., 2018) and horses (Gondim et al., 2007; Miranda et al., 2014; Soares et al., 2014) since its initial description in the 1990s.

It should be noted that there is a debate over the effectiveness of the typical LMS protocol in detecting small changes in aerobic capacity, which may be resulting from differences in the LMT protocol or the mathematical methods used. Specific studies have provided insight into this matter. Although LMT is commonly applied in conditioning program, using traditional LMS to assess aerobic fitness is still debatable (Messias et al., 2017b). After 6 weeks of conditioning, eight well-trained endurance runners showed an increase in their maximum oxygen intake ( ${\dot{V}O}_{2}$ _MAX_), but no changes in their LMS were detected (Carter et al., 1999). Similarly, in swimmers, changes in LMS were observed after 4 weeks of training but not between four and 12 weeks (Campos et al., 2014). The present study observed no changes in the typical LMS after the training. Alternative mathematical approaches used herein could detect an improvement in aerobic fitness, as was the case of alpha angle, α:β ratio, and AUC_POSTLMS_. The results indicate that horses have a better aerobic capacity caused by conditioning, as shown by lower lactate accumulation during the incremental phase. Furthermore, the low bias associated with the high ICC value found between LMS_S_ and LMS_V_ showed good practical applicability of visual inspection for determining LMS in horses. This finding may be attributed to the low sensitivity of the conventional LMS protocol in identifying small changes in aerobic capacity (Carte et al., 1999), the influence of the LMT protocol adopted (Ribeiro et al., 2009; Zagatto et al., 2014), or the mathematical method used (Messias et al., 2017a; Messias et al., 2017b). Despite the controversy over the influence of different methodologies applied for phase I (hyperlactatemia) (Smith et al., 2002; Zagatto et al., 2014), it is known that both phase II (recovery) and phase III (IET) can affect the LMS (Carter et al., 1999; Ribeiro et al., 2009).

Among the conventional methods for determining the LMS are second-degree polynomial regression, spline regression and visual inspection. As demonstrated, LMS_P_ showed the highest values, followed by LMS_S_ and LMS_V,_ and finally LMS_BI._. To the best of our knowledge, the determination of LMS_BI_ by the method used is unprecedented in horses and differs from other studies in which the bi-segmented adjustment is followed by empirically inspection of the curve to determine the point of interest (Zagatto et al., 2008; Ferraz et al., 2022). Regarding the spline method (called B-spline), it differs from previous approaches (Tegtbur et al., 1993; Carte et al., 1999) since the parameterization carried out (see item 2.6.2) provides greater numerical stability (for more details, see Perperoglou et al., 2019). With respect to the second-degree polynomial regression method, it is frequently reported in studies on the use of LMT in humans (Ribeiro et al., 2009; Beck et al., 2012; Zagatto et al., 2014) and horses (Soares et al., 2014; Santos et al., 2022), and therefore was chosen as the standard for determining the LMS (LMS_P_). This method was also employed to determine the speed corresponding to lactate threshold obtained in the curve between [Lac]:speed ratio (*y*-axis) and speed (*x*-axis) in dogs (Ferraz et al., 2022).

Some studies reported the use of third-degree polynomial regression applied to LMT in runners, resulting in a low mean bias (0.04) and a high ICC (0.956) in relation to MLSS (Wahl et al., 2018). It is important to highlight that in the present study the LMS was not only employed to assess conditioning, but also served as a basis for determining the training load to be used, similarly to the study carried out by De Maré et al. (2022). In another study conducted by our research group (Santos et al., 2022), although LMS was applied in both training prescription and conditioning assessment, only the LMS_P_ was calculated.

It was found that the visual method for evaluating the curve produced by LMT has good usability, as indicated by an ICC >0.9 for the LMS_S_, LMS_P_, and LMS_V_ methods. These methods showed high agreement with more robust mathematical methods. Studies conducted on humans (Wahl et al., 2018) and dogs subjected to incremental progressive standardized exercise tests (Ferraz et al., 2022) reported low biases between the visual method and MLSS, suggesting that the method is applicable in different scenarios.

One of the latest methods to assess the curve produced by LMT is the utilization of aerobic window (AW), which is considered a significant advancement in the training of athletic horses. The AW approach enables coaches to work with a range of speeds instead of a fixed value, providing greater flexibility and more precise load adjustment during the training period. A study displayed that the 40° angle found showed better mathematical robustness in determining the AW in horses (De Maré et al., 2022). This study served as a basis for developing new approaches regarding the LMT performed in the present study. Although the methodological description of AW raises doubts about the way in which the angles are obtained, in our study a modified methodology was applied, resulting in the obtainment of three angles denominated α, β and ω (see item 2.8), whose average values suggest an evolution of the animals after conditioning, given the reduction observed in angle α, which indicates reduced lactate accumulation during the incremental phase possibly attributable to an improvement in the aerobic metabolism. Consequently, the curve becomes “flattened”, with a consequent decrease in α (Figure 7). An increase in angle β would indicate a more abrupt lactate drop during the active recovery phase, that is, an increase in lactate clearance capacity. However, in this current study no significant changes resulting from conditioning were detected. Although not significant, the reduction in α:β ratio associated with the increase in angle ω suggests an expansion of the AW and consequently the speed range, where there is a steady state between lactate production and removal (De Maré et al., 2022).

In order to analyze the accumulation of blood lactate during the LMT, we calculated the AUC using a standard method for assessing lactate concentration in drugs pharmacokinetic studies (Purves, 1992; Tian et al., 2020) with applications in lactate concentration curves for monitoring foals in critical clinical condition (Wilkins et al., 2015) and determining their conditioning by LMT curves (De Maré et al., 2022). The most used way to determine AUC is the trapezoidal method, which has mathematical simplicity and easy application (Purves, 1992; Wilkins et al., 2015). Nevertheless, this method can yield discrepant values, ​​given the high percentages of the mean squared error (Purves, 1992). To address the issue, this study utilized spline regression to determine AUC. This method is more flexible in modeling the data and does not assume any possible trends, making it suitable for calculating total or partial AUC. This contrasts with the approach suggested by De Maré et al. (2022), who calculated the AUC using data adjusted with second-degree polynomial regression. The present study, on the other hand, used actual data obtained during the LMT to calculate the AUC. The AUC values found corroborate those obtained by the angular method since they show differences in the AUC_POSTLMS_ values, which implies lower lactate accumulation during the incremental phase after training, as indicated by the change in angle α. No significant changes were observed in AUC_TOTAL_ and AUC_PRELMS_, also in agreement with the results of angle β, demonstrating similarity between the hyperlactatemia and dynamic recovery phases after conditioning.

The results obtained in the AUC analysis correspond to those obtained by the angular method. The differences observed in AUC_POSTLMS_ after the conditioning period indicate lower lactate accumulation during the incremental phase, as indicated by the reduction in angle α. No significant changes were found in AUC_TOTAL_ and AUC_PRELMS_, demonstrating similarities between the hyperlactatemia and dynamic recovery phases in animals before and after conditioning.

This study found that lower lactate accumulation during the incremental phase increased the aerobic window. This detection was shown by a reduction in angle α and an increase in ω, which have a negative correlation (r = −0.98; *p* < 0.001). However, angle β was not sensitive enough to evaluate the initial phases of the exercise due to the lack of positive correlations with any of the variables. The AUC_PRELMS_ had a strong positive correlation with AUC_TOTAL_, highlighting the importance of hyperlactatemia and dynamic recovery phases during exercise.

Although our work is innovative, it is important to recognize its limitations. The present trial can be considered a before-and-after study (Bou et al., 2024) since there was no non-training control. It should be noted that the horses in the study were kept under a pasture regime, which ensured voluntary-spontenous exercise. The horses could engage in regular equine behaviors such as moving, socializing, and maintaining their overall welfare. It should be highlighted that this study offers new insights into endurance training on the lactate minimum speed approach. Further experiments with larger sample sizes are needed to examine the effects of traditional and non-traditional LMT protocols for endurance training in horses. Furthermore, it was not possible to determine the degree of reliability of the different LMS determination methods in predicting the MLSS, as this value was not determined. Possible changes in MLSS resulting from conditioning would confirm the improvement in the aerobic capacity of the animals, producing more robust evidence about the sensitivity of the new approaches proposed for assessing the conditioning of horses.

## 5 Conclusion

The reductions observed in angle α, α:β ratio and AUC_POSTLMS_ as a result of conditioning demonstrated greater sensitivity of these variables in relation to LMS in evaluating the physiological responses promoted by training. Finally, it can be concluded that the LMT is an efficient test with wide practical applications that can be determined using different mathematical approaches.

## Data Availability

The raw data supporting the conclusion of this article will be made available by the authors, without undue reservation.
